# Supplementary material for: Asymmetric Synthesis of Quaternary Hydantoins via a Palladium-Catalyzed Aza-Heck Cyclization
Source: J Am Chem Soc. 2025 Nov 14;147(49):44692–8. doi: 10.1021/jacs.5c16022 (PMC12703750; doi:10.1021/jacs.5c16022)
Supplement: Supplementary file 2 [file ja5c16022_si_002.zip › All NMR FID Files/S60/2/pdata/1/email_EM04-032-1_2_1.pdf]

C13APT2048 CDCl3 /opt/nmrdata emeck 2

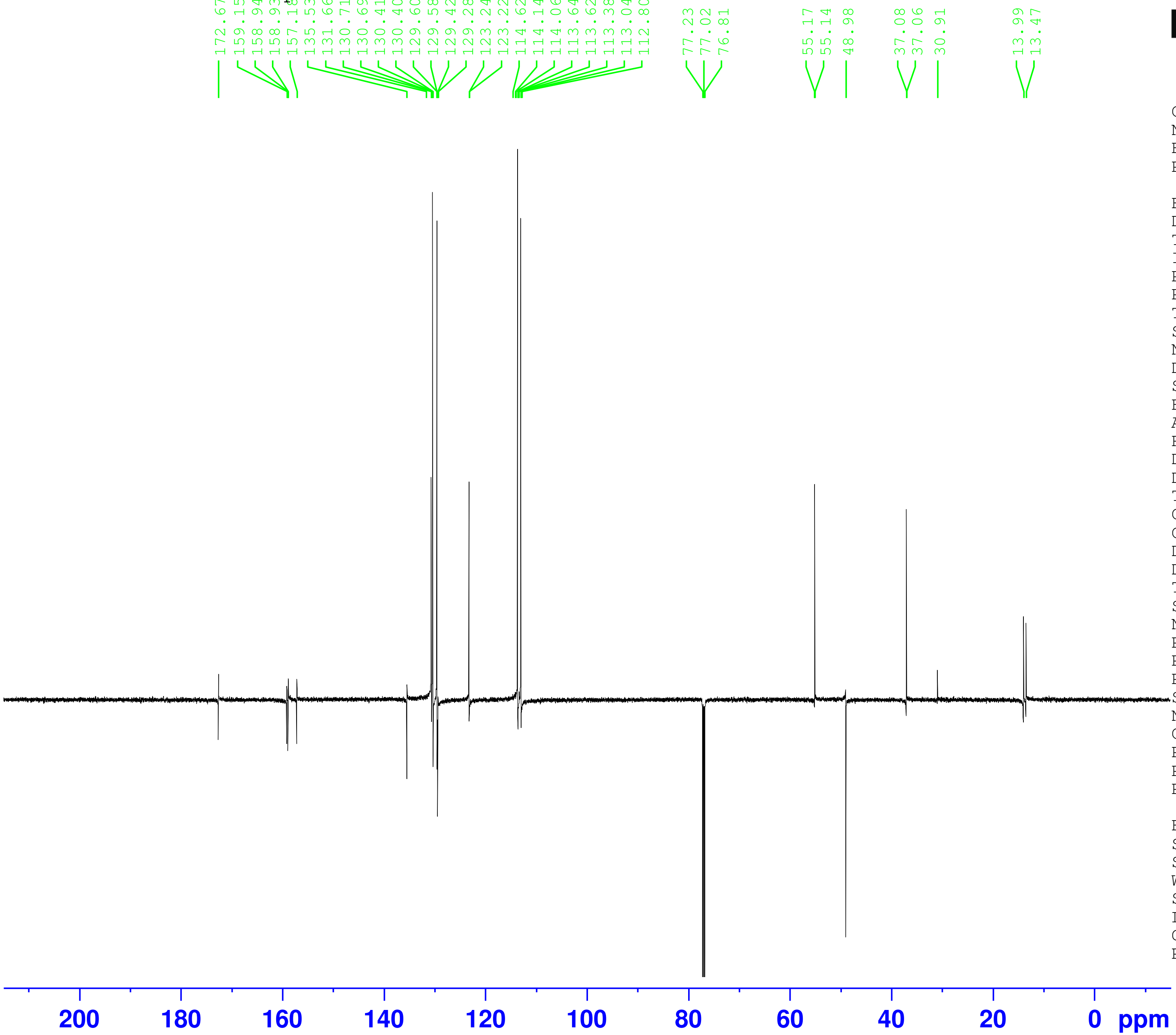

BRUKER

Current Data Parameters

NAME	EM04-032-1	
EXPNO	2	
PROCNO	1	

F2 - Acquisition Parameters

Date_	20230320	
Time	20.28	h
INSTRUM	spect	
PROBHD	Z114607_0069 (	
PULPROG	jmod.acqt0	
TD	65536	
SOLVENT	CDCl3	
NS	2048	
DS	4	
SWH	34722.223	Hz
FIDRES	1.059638	Hz
AQ	0.9437184	sec
RG	2050	
DW	14.400	usec
DE	7.38	usec
TE	300.0	K
CNST2	145.0000000	
CNST11	1.0000000	
D1	5.00000000	sec
D20	0.00689655	sec
TD0	1	
SFO1	150.9656791	MHz
NUC1	13C	
P1	12.00	usec
P2	24.00	usec
PLW1	110.00000000	W
SFO2	600.3224013	MHz
NUC2	1H	
CPDPRG[2	waltz16	
PCPD2	70.00	usec
PLW2	26.00000000	W
PLW12	0.65967000	W

F2 - Processing parameters

SI	32768	
SF	150.9505840	MHz
WDW	EM	
SSB	0	
LB	1.00	Hz
GB	0	
PC	1.40	
